# Clinical course of nontuberculous mycobacterial pulmonary disease in patients with rheumatoid arthritis

**DOI:** 10.1186/s42358-024-00357-z

**Published:** 2024-03-15

**Authors:** Nakwon Kwak, Jinyoung Moon, Joong-Yub Kim, Jun Won Park, Jae-Joon Yim

**Affiliations:** 1 Division of Respiratory and Critical Care Medicine, Department of Internal Medicine, Seoul National University College of Medicine, 101 Daehak-Ro, Jongo-Gu, Seoul, 03080 South Korea; 2 Chaum Life Center, CHA University School of Medicine, Seoul, South Korea; 3 Division of Rheumatology, Department of Internal Medicine, Seoul National University College of Medicine, Seoul, South Korea

**Keywords:** Mortality, Nontuberculous mycobacteria, Prognosis, Pulmonary disease, Rheumatoid arthritis

## Abstract

**Objectives:**

The impact of rheumatoid arthritis (RA) on nontuberculous mycobacterial pulmonary disease (NTM-PD) has not been well established. In this study, we investigated the clinical course of NTM-PD in patients with RA and the impact of RA on the prognosis of NTM-PD.

**Methods:**

We analyzed patients who developed NTM-PD after being diagnosed with RA from January 2004 to August 2023 at a tertiary referral hospital in South Korea. The patient’s baseline characteristics, clinical course, and prognosis were evaluated. An optimal matching analysis was performed to measure the impact of RA on the risk of mortality.

**Results:**

During the study period, 18 patients with RA [median age, 68 years; interquartile range (IQR) 59–73; female, 88.9%] developed NTM-PD. The median interval between RA diagnosis and subsequent NTM-PD development was 14.8 years (IQR, 8.6–19.5). At a median of 30 months (IQR, 27–105) after NTM-PD diagnosis, 10 of 18 (55.6%) patients received anti-mycobacterial treatment for NTM-PD and 5 (50.0%) patients achieved microbiological cure. When matched to patients with NTM-PD but without RA, patients with both RA and NTM-PD had a higher risk of mortality (adjusted hazard ratio, 8.14; 95% confidence interval, 2.43–27.2).

**Conclusion:**

NTM-PD occurring after RA is associated with a higher risk of mortality than NTM-PD in the absence of RA.

**Supplementary Information:**

The online version contains supplementary material available at 10.1186/s42358-024-00357-z.

## Background

Nontuberculous mycobacteria (NTM), comprising more than 200 mycobacterial species other than *Mycobacterium tuberculosis* and *M. leprae*, are ubiquitous organisms found in soil, dust, and municipal water [1]. NTM can lead to chronic infections in humans, with pulmonary disease (PD) being the most common presentation [1]. Most patients with NTM-PD present with cough, sputum production, fatigue, malaise, dyspnea and weight loss [2]. The epidemiological importance of NTM-PD has been emphasized during the past few decades. In South Korea, the annual prevalence of NTM-PD increased approximately five-fold from 2010 to 2021 [3].

Pre-existing respiratory comorbidities significantly increase the risk of NTM-PD. Conditions such as bronchiectasis, chronic obstructive pulmonary disease, interstitial lung disease (ILD), or previous tuberculosis infection have been associated with the development of NTM-PD [4]. Although structural lung disease increases the susceptibility to NTM infection, the use of immunosuppressive agents further enhances the risk of NTM-PD [5, 6]. Exposure to anti-tumor necrosis factor agents has been linked to an increased risk of both tuberculosis and NTM infection. Moreover, the administration of systemic steroids raises the risk of NTM infection [5].

Rheumatoid arthritis (RA) is a chronic autoimmune disease affecting 0.27–1.85% of the general population in South Korea [7]. Several studies have demonstrated a close association between RA and NTM-PD. The structural lung abnormalities in RA can predispose individuals to NTM acquisition [8]. Restriction of the T-cell repertoire in patients with RA impairs the ability to respond to NTM [8]. Additionally, the immunomodulation induced by biologic and non-biologic disease-modifying anti-rheumatic drugs (DMARDs) contributes to the development of NTM-PD [5]. As a result, the incidence of NTM-PD in patients with RA is higher than that in the general population [9]. According to a population-based study, the risk of developing NTM-PD in patients with RA was 4.86 times higher than in the general population, resulting in an incidence rate of 41.6 cases per 100,000 person-years [10].

Although the association between RA and the development of NTM-PD has been confirmed, the impact of RA on the course of NTM-PD is not well-established. In some studies, the type of DMARDs used after the diagnosis of NTM-PD did not affect clinical or radiographic deterioration [11, 12], and RA did not increase the risk of mortality in patients with NTM-PD [13]. Notably, however, these findings were obtained from retrospective studies. Additional data accumulation is warranted to gain a more comprehensive understanding of how RA affects the clinical course of NTM-PD.

In this study, we investigated the clinical course of NTM-PD in patients with RA and the impact of RA on the prognosis of NTM-PD at a tertiary referral center in South Korea.

## Methods

### Study design

The study has two arms. First, the clinical course of NTM-PD in patients with RA was analyzed using the detailed data obtained from patients initially diagnosed with RA who were subsequently diagnosed with NTM-PD. Second, the impact of RA on the prognosis of NTM-PD was determined based on the case-control study, with the control being patients with NTM-PD but without RA. The study protocol was approved by the Institutional Review Board at Seoul National University Hospital (No. 2309-032-1463), and the need to obtain informed consent was waived. This study was performed in accordance with the Declaration of Helsinki.

### Patient selection

We screened patients with RA who were aged ≥ 18 years and fulfilled the 1987 American College of Rheumatology criteria or the 2010 American College of Rheumatology/European League Against Rheumatism criteria for a diagnosis of RA [14, 15] from 1 January 2004 to 31 August 2023 at Seoul National University Hospital. Among these patients, we retrospectively analyzed those who met the diagnostic criteria for NTM-PD (having all of the following: pulmonary or systemic symptoms, nodular or cavitary lesions on chest radiographs, and positive culture results from at least two sputum samples or at least one bronchial wash or lavage) as suggested by the American Thoracic Society/European Respiratory Society/European Society of Clinical Microbiology and Infectious Diseases and the Infectious Diseases Society of America [1].

### Data collection

We collected the patients’ demographic and clinical data, including age, sex, body mass index, acid-fast bacilli (AFB) smear results, mycobacterial species, and the presence of cavities on chest computed tomography scans at the time of NTM-PD diagnosis. Additionally, we recorded the presence of rheumatoid factor or anti-citrullinated peptide antibodies at the time of RA diagnosis, the duration of RA, and the DMARDs used for at least 1 month before and after NTM-PD diagnosis. To determine the incidence rate of NTM-PD after the onset of RA, we measured the duration of follow-up until the occurrence of NTM-PD or until the last visit, which was censored on 31 August, 2023. Once anti-mycobacterial treatments were initiated, we measured the time to the start of anti-mycobacterial treatment, the administered drugs, and the treatment outcomes.

### Definitions

The progression of NTM-PD was determined by the initiation of anti-mycobacterial treatment as decided by the attending physician [16]. Microbiological cure of NTM-PD was defined as three or more consecutive negative cultures of respiratory samples after achieving culture conversion until the completion of anti-mycobacterial treatment [17]. Information regarding the date of death was obtained from the Ministry of the Interior and Safety of South Korea, and the time to death was calculated as the duration from the date of NTM-PD diagnosis to the date of death.

### Case-control study

To assess the impact of RA on the clinical course of NTM-PD, we matched cases [patients with both RA and NTM-PD, referred to as RA (+) NTM-PD] to controls [patients with NTM-PD but without RA who were diagnosed from 1 January 2011 to 31 August 2023, referred to as RA (−) NTM-PD] from a prospective cohort of patients with NTM-PD in our institution [16, 18–21]. The cases and controls were matched in a 1:5 ratio. NTM-PD progression and mortality were compared between the RA (+) NTM-PD and RA (−) NTM-PD groups.

### Statistical analysis

Data are summarized as median with interquartile range (IQR) for continuous variables and as proportion for categorical variables. Optimal matching using the network flow methodology was performed to match the cases and controls for age, sex, body mass index, AFB smear results, mycobacterial species, and presence of cavities [22]. The Kruskal–Wallis test and Fisher’s exact test were used for continuous and categorical variables, respectively. Survival data were analyzed using Kaplan–Meier analysis with a log-rank test and multivariable Cox proportional hazard regression. Variables with a *P*-value of < 0.20 in the univariate analysis were entered into the multivariate analysis. All statistical analyses were performed using Stata version 17.0 (StataCorp, College Station, TX, USA).

## Results

### Patient characteristics

During the study period, 9,908 patients were diagnosed with RA, and 18 of those patients developed NTM-PD. The incidence rate of NTM-PD after RA diagnosis was 21.8 per 100,000 person-years. The patients’ median age was 68 years (IQR, 59–73), and 16 (88.9%) patients were female. The median interval from RA diagnosis to subsequent NTM-PD development was 14.8 years (IQR, 8.6–19.5) (Table 1). Eight (44.4%) patients had RA-associated ILD before the diagnosis of NTM-PD. At the time of NTM-PD diagnosis, glucocorticoids were administered to 14 (77.8%) patients at a median daily prednisolone-equivalent dose of 4.7 mg (IQR, 2.5–7.5). Methotrexate (17 patients, 94.4%) was the most commonly used DMARD, followed by hydroxychloroquine (12 patients, 66.7%) and sulfasalazine (11 patients, 61.1%). Biologic agents were used in four patients (adalimumab, *n* = 2; infliximab, *n* = 1; and tocilizumab, *n* = 1). The time to NTM-PD development was not different between patients treated with biologics (median, 14.5 years; IQR, 10.6–17.3) and those not treated with biologics (median, 14.8 years; IQR, 8.6–20.9) (*P* = 0.632).

**Table 1:** Baseline characteristics of the study population

Characteristics	Value (*N* = 18)
Age, years, median (IQR)	68 (59–73)
Female, N (%)	16 (88.9)
Body mass index, median (IQR)	20.6 (16.2–21.3)
RA duration, years, median (IQR)	14.8 (8.6–19.5)
Rheumatoid factor positive, N (%)	14 (77.8)
Anti-CCP antibody positive, N (%)	9 (50.0)
RA-associated interstitial lung disease, N (%)	8 (44.4)
Conventional synthetic DMARDs before NTM-PD diagnosis	
Methotrexate	17 (94.4)
Hydroxychloroquine	12 (66.7)
Sulfasalazine	11 (61.1)
Leflunomide	5 (27.8)
Tacrolimus	2 (11.1)
Biologics used before NTM-PD diagnosis	
Infliximab	1 (5.6)
Adalimumab	2 (11.1)
Tocilizumab	1 (5.6)
AFB smear positivity, N (%)	4 (22.2)
Mycobacterial species, N (%)	
*M. avium*	10 (55.6)
*M. intracellulare*	6 (33.3)
*M. abscessus* subspecies *abscessus*	1 (5.6)
*M. abscessus* subspecies *massiliense*	1 (5.6)
Presence of cavity, N (%)	6 (33.3)

RA, rheumatoid arthritis, anti-CCP, anti-cyclic citrullinated peptide; DMARDs, disease modifying anti-rheumatic drugs, NTM-PD, nontuberculous mycobacterial pulmonary disease

### Clinical course of NTM-PD

After the diagnosis of NTM-PD, 16 of 18 (88.9%) patients received DMARDs for RA. Biologic agents were used for two (11.1%) patients. Glucocorticoids were administered to 16 (88.9%) patients with a median monthly prednisolone-equivalent dose of 150 mg (IQR, 75–318). During the median follow-up of 60 months (IQR, 8-102), eight (44.4%) patients were followed up without anti-mycobacterial treatment. This was dictated by the absence of symptom worsening or radiographic deterioration attributable to NTM-PD. However, ten of 18 (55.6%) patients received anti-mycobacterial treatment for NTM-PD at a median interval of 30 months (IQR, 27–105) after NTM-PD diagnosis. This was due to disease progression, with two patients experiencing symptomatic worsening, three experiencing radiographic deterioration, and five experiencing both. All patients were treated with macrolide-based regimens. Three patients received both amikacin and clofazimine. Five of 10 patients achieved microbiological cure for NTM-PD. During a median follow-up period of 40 months (IQR, 19–105), six patients died (four in patients who received anti-mycobacterial treatment and two in patients who did not receive anti-mycobacterial treatment), and all deaths were attributed to respiratory disease (pneumonia, *n* = 4; RA-ILD exacerbation, *n* = 1; and progression of NTM-PD, *n* = 1). Detailed information on the patients’ clinical outcomes following NTM-PD diagnosis is provided in Table 2.

**Table 2:** Detailed clinical information of each patient after diagnosis of NTM-PD

No.	Sex/Age	BMI	RA-ILD	Drugs used for RA after NTM-PD diagnosis	Species	Cavity	Treatment for NTM-PD	Treatment outcome	Death during follow-up period	Cause of death
1	F/69	21.9	Yes	Methotrexate, hydroxychloroquine, tacrolimus, corticosteroid	*M. avium*	No	Clarithromycin, rifampicin, isoniazid	Cured	Yes	Bacterial pneumonia
2	F/77	16.2	No	Methotrexate, rituximab, corticosteroid	*M. massiliense*	No	No	N/A	No	N/A
3	F/59	15.0	No	Methotrexate, leflunomide, sulfasalazine, corticosteroid	*M. avium*	No	No	N/A	No	N/A
4	F/74	21.1	No	Hydroxychloroquine, corticosteroid	*M. intracellulare*	Yes	No	N/A	No	N/A
5	F/73	20.6	No	Methotrexate, leflunomide, corticosteroid	*M. abscessus*	No	No	N/A	Yes	Bacterial pneumonia
6	F/58	22.3	No	Methotrexate, hydroxychloroquine, sulfasalazine, corticosteroid	*M. avium*	No	No	N/A	No	N/A
7	F/70	19.4	No	Methotrexate, corticosteroid	*M. intracellulare*	No	Azithromycin, ethambutol	Failed	No	N/A
8	F/67	15.4	Yes	Methotrexate, hydroxychloroquine	*M. intracellulare*	Yes	Clarithromycin, ethambutol, rifampicin	Cured	Yes	Bacterial pneumonia
9	F/79	20.9	Yes	Hydroxychloroquine, corticosteroid	*M. intracellulare*	Yes	Azithromycin, ethambutol, clofazimine, amikacin	Cured	No	N/A
10	F/40	21.2	Yes	Methotrexate, hydroxychloroquine, sulfasalazine, corticosteroid	*M. intracellulare*	Yes	Azithromycin, ethambutol, rifampicin, clofazimine	Cured	No	N/A
11	F/67	20.3	Yes	Sulfasalazine, corticosteroid	*M. avium*	No	Azithromycin, ethambutol, clofazimine, amikacin	Failed	Yes	Worsening of RA-ILD
12	F/65	23.8	No	Methotrexate, tacrolimus	*M. avium*	No	Azithromycin, ethambutol, rifampicin	Failed	No	N/A
13	F/56	15.1	No	Hydroxychloroquine, tacrolimus, sulfasalazine, rituximab, corticosteroid etanercept	*M. avium*	No	Clarithromycin, ethambutol, levofloxacin	Cured	No	N/A
14	F/57	20.6	No	Methotrexate, leflunomide, sulfasalazine, corticosteroid	*M. intracellulare*	No	No		No	N/A
15	F/84	21.3	Yes	Corticosteroid	*M. avium*	No	No		No	N/A
16	M/66	18.1	No	Corticosteroid	*M. avium*	Yes	No		Yes	Bacterial pneumonia
17	F/68	16.0	Yes	Sulfasalazine, corticosteroid	*M. avium*	No	Azithromycin, ethambutol, amikacin, moxifloxacin	Failed	Yes	Worsening of NTM-PD
18	M/72	22.3	Yes	Methotrexate, leflunomide, sulfasalazine, corticosteroid, adalimumab, abatacept	*M. avium*	Yes	Azithromycin, ethambutol, rifampicin, clofazimine, amikacin	Failed	No	N/A

RA-ILD, rheumatoid arthritis-associated interstitial lung disease; NTM-PD, nontuberculous mycobacterial pulmonary disease; N/A, not applicable

### Prognostic impact of RA on NTM-PD

**Fig. 1:**
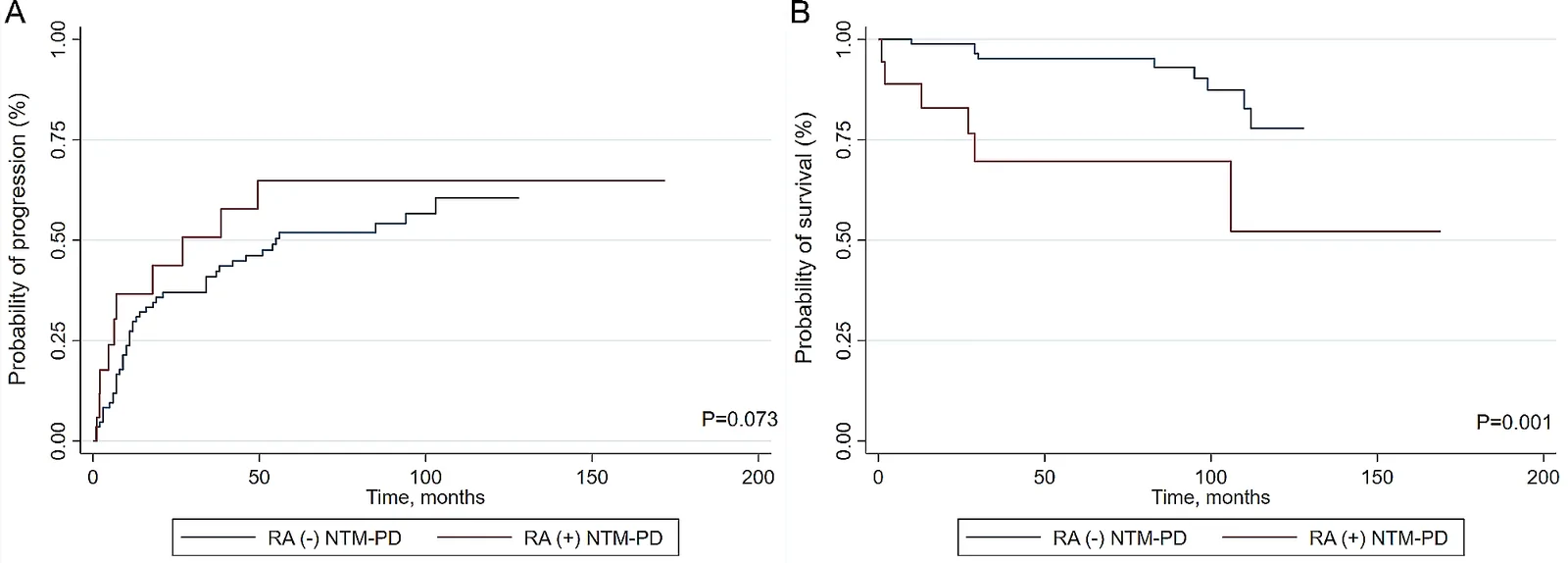
Kaplan–Meier curves for (**A**) time to disease progression and (**B**) time to death

After optimal matching, every patient with RA (+) NTM-PD was paired with 90 patients with RA (−) NTM-PD. The characteristics of the two groups are presented in Table 3. The time to NTM-PD progression was not different between the two groups (log-rank *P* = 0.073), and the presence of RA did not affect the progression of NTM-PD (Table 4). The microbiological cure rate also showed no difference between the RA (+) NTM-PD and RA (−) NTM-PD groups (50.0% and 53.1%, respectively; *P* > 0.999). However, the patients in the RA (+) NTM-PD group had worse survival rates than those in the RA (−) NTM-PD group (log-rank *P* = 0.001). According to the multivariate Cox proportional hazard analysis, patients with RA and NTM-PD had a higher risk of mortality compared to those with NTM-PD alone (adjusted hazard ratio, 8.14; 95% confidence interval, 2.43–27.2) (Table 5). The Kaplan–Meier plots for disease progression and survival are shown in Fig. 1.

**Table 3:** Characteristics of study group and matched control group

	RA (+) NTM-PD (*N* = 18)	RA (-) NTM-PD (*N* = 90)	*P*-value
Age, years, median (IQR)	68 (59–73)	68 (60–73)	0.830
Female, N (%)	16 (88.9)	80 (88.9)	> 0.999
Body mass index, median (IQR)	20.6 (16.2–21.3)	20.0 (18.0–21.0)	0.642
Comorbidities, N (%)			
History of tuberculosis	2 (11.1)	336 (40.0)	0.028
Diabetes	3 (16.7)	5 (5.6)	0.127
Chronic obstructive pulmonary disease	0	4 (4.4)	0.999
Malignancy	0	11 (12.2)	0.205
Presence of cavity, N (%)	6 (33.3)	30 (33.3)	> 0.999
AFB smear positivity, N (%)	4 (22.2)	23 (25.6)	> 0.999
*M. avium* complex, N (%)	16 (88.9)	77 (85.6)	> 0.999
Administration of anti-mycobacterial treatment, N (%)	10 (55.6%)	50 (55.6)	> 0.999
Microbiological cure, N (%)	5/10 (50.0%)	26/50 (53.1%)	> 0.999

AFB, acid fast bacilli; IQR, interquartile range; NTM-PD, nontuberculous mycobacterial pulmonary disease; RA, rheumatoid arthritis

**Table 4:** Factors associated with disease progression in patients with nontuberculous mycobacterial pulmonary disease

	Univariate analysis		Multivariate analysis	
	HR (95% CI)	*P*-value	adjusted HR (95% CI)	*P*-value
Age, years	0.98 (0.95–1.01)	0.190	0.97 (0.94–0.99)	0.038
Male sex	1.73 (0.78–3.86)	0.180	0.91 (0.36–2.32)	0.846
Body mass index, kg/m^2^	0.97 (0.86–1.09)	0.571		
Former or current smoking	0.96 (0.35–2.67)	0.941		
Comorbidities				
History of tuberculosis	1.20 (0.70–2.07)	0.500		
Diabetes	1.06 (0.39–2.92)	0.917		
Chronic obstructive pulmonary disease	1.37 (0.43–4.38)	0.360		
Malignancy	0.89 (0.38–2.08)	0.792		
Rheumatoid arthritis	1.42 (0.72–2.82)	0.319		
AFB smear positivity	2.03 (1.13–3.64)	0.018	2.65 (1.41-5.00)	0.003
*M. abscessus* versus *M. avium* complex	1.46 (0.73–2.89)	0.283		
Presence of cavity	2.23 (1.28–3.85)	0.004	2.45 (1.30–4.60)	0.006

AFB, acid fast bacilli; HR, hazard ratio

**Table 5:** Factors associated with mortality in patients with nontuberculous mycobacterial pulmonary disease

	Univariate analysis		Multivariate analysis	
	HR (95% CI)	*P*-value	adjusted HR (95% CI)	*P*-value
Age, years	1.07 (1.01–1.14)	0.044	1.09 (1.01–1.17)	0.023
Male sex	6.87 (2.23–21.1)	0.001	6.39 (1.16–35.4)	0.034
Body mass index, kg/m^2^	0.95 (0.76–1.19)	0.650		
Smoking	2.29 (0.52–10.2)	0.277		
Comorbidities				
History of tuberculosis	0.89 (0.30–2.60)	0.831		
Diabetes	4.06 (1.12–14.7)	0.033	0.37 (0.06–2.13)	0.264
Chronic obstructive pulmonary disease	2.61 (0.58–11.8)	0.210		
Malignancy	0.52 (0.07–3.94)	0.524		
Rheumatoid arthritis	4.28 (1.52–12.1)	0.006	8.14 (2.43–27.2)	0.001
AFB smear positivity	2.08 (0.69–6.22	0.192	1.15 (0.34–3.88)	0.816
*M. abscessus* versus *M. avium* complex	0.86 (0.19–3.84)	0.849		
Presence of cavity	3.05 (1.09–8.49)	0.032	2.13 (0.53–8.59)	0.288

AFB, acid fast bacilli; HR, hazard ratio

## Discussion

In this study, we investigated the clinical course of patients initially diagnosed with RA who later developed NTM-PD. Most patients continued to receive DMARDs and glucocorticoids for RA after the diagnosis of NTM-PD. More than half of the patients received anti-mycobacterial treatment for NTM-PD, and among them, 50% of the patients achieved microbiological cure. When RA coexisted with NTM-PD, the risk of mortality was higher than that in the absence of RA. The primary causes of death were predominantly related to respiratory complications.

With the increasing use of various biologics in clinical practice, there is a growing interest in the development of mycobacterial infection in patients with connective tissue diseases [23–25]. RA has been actively studied in this context, and several studies have demonstrated an increased risk of NTM-PD in patients with RA [9, 26]. However, few studies have provided insights into the clinical course of NTM-PD after its occurrence in patients with RA. Therefore, in this study, we rigorously focused on the clinical course of NTM-PD in 18 patients who were diagnosed with both RA and NTM-PD.

All 18 patients were diagnosed with NTM-PD approximately 15 years after their initial diagnosis of RA. Considering the incidence rate of 21.8 cases per 100,000 person-years in this study, the occurrence of NTM-PD in patients with RA is relatively uncommon. Although most patients had a history of prolonged exposure to glucocorticoids and DMARDs prior to NTM-PD development, their age at the time of NTM-PD diagnosis, distribution of mycobacterial species, and AFB smear positivity were comparable with those of patients with NTM-PD but without RA in South Korea [27, 28]. These results imply that the clinical phenotypes of NTM-PD are not markedly different between patients with and without RA.

The decision to initiate anti-mycobacterial treatment is individualized based on the clinical contexts. Approximately one-third of patients with non-cavitary nodular bronchiectatic NTM-PD can achieve spontaneous culture conversion without treatment [29]. However, about half of patients eventually require treatment for NTM-PD due to symptomatic worsening or radiographic aggravation [30]. In this study, anti-mycobacterial treatment was administered in 10 of 18 patients. The interval between diagnosis and treatment for NTM-PD was 30 months, which was comparable to that of patients without RA. Moreover, once anti-mycobacterial treatment was initiated, the treatment outcomes were also comparable to those in patients without RA.

While RA itself and the subsequent use of immunosuppressive agent increase the risk of infection [31], our findings suggest that the presence of RA and the use of immunosuppressive agents do not dictate the severity or progression of NTM-PD. This disparity may be explained by the distinct immune mechanism underlying systemic NTM infection and NTM-PD, a localized infection [32]. NTM-PD appears to be more influenced by local immunity than systemic immunity [32]. Indeed, the impact of corticosteroid on the development of NTM-PD has been more extensively studied with inhaled corticosteroid than with systemic corticosteroid [33, 34]. These observations are further supported by another Korean study that showed no discernible difference in comorbidities or the use of immunosuppressive agents between patients with progressive NTM-PD and patients with stable NTM-PD [35].

Although RA did not impact the progression of NTM-PD, RA increased the risk of mortality in patients with NTM-PD. Male sex, older age, and the presence of cavity are established risk factors for mortality [18]. In our study, the impact of RA on mortality exceeded that of these variables. This finding contradicts a Japanese study showing that RA did not increase the risk of mortality in patients with NTM-PD [13]. Importantly, our study showed that only one of six deaths was attributed to the progression of NTM-PD; the other five deaths were directly related to respiratory diseases, including pneumonia and exacerbation of ILD. These results suggest that the co-existence of ILD or the subsequent infection due to immunosuppressive agents, rather than NTM-PD itself, increased the risk of respiratory complications, which may then lead to death in patients with NTM-PD. However, due to the small sample size in this study, the impact of ILD or immunosuppressive agents should be interpreted with caution.

This study has several limitations. First, the sample size was small because of the relative rarity of both NTM and RA. However, we mitigated this limitation through optimal matching. Second, we were unable to comprehensively adjust for RA disease activity, which is an inherent limitation of retrospective studies. Third, because the cause of death in patients with RA (−) NTM-PD was not fully available, the risk of mortality attributed to NTM-PD could not be compared.

In conclusion, NTM-PD occurring after RA is associated with a higher risk of mortality than NTM-PD without RA, and respiratory diseases are a predominant cause of death.

## Electronic supplementary material

Below is the link to the electronic supplementary material.

Supplementary Material 1

## Data Availability

The dataset used are available from the corresponding author on reasonable request.
